# *Staphylococcus aureus* and the Cutaneous Microbiota Biofilms in the Pathogenesis of Atopic Dermatitis

**DOI:** 10.3390/microorganisms7090301

**Published:** 2019-08-29

**Authors:** Enea Gino Di Domenico, Ilaria Cavallo, Bruno Capitanio, Fiorentina Ascenzioni, Fulvia Pimpinelli, Aldo Morrone, Fabrizio Ensoli

**Affiliations:** 1 Clinical Pathology and Microbiology, San Gallicano Dermatologic Institute, IRCCS, 00144 Rome, Italy; 2 Division of Dermatology, San Gallicano Dermatologic Institute, IRCCS, 00144 Rome, Italy; 3 Department of Biology and Biotechnology C. Darwin, University of Rome Sapienza, 00161 Rome, Italy; 4 Scientific Director San Gallicano Dermatological Institute IRCCS, 00144 Rome, Italy

**Keywords:** atopic dermatitis, *Staphylococcus aureus*, biofilm, biotherapy, skin microbiome, cytokines

## Abstract

Biofilm is the dominant mode of growth of the skin microbiota, which promotes adhesion and persistence in the cutaneous microenvironment, thus contributing to the epidermal barrier function and local immune modulation. In turn, the local immune microenvironment plays a part in shaping the skin microbiota composition. Atopic dermatitis (AD) is an immune disorder characterized by a marked dysbiosis, with a sharp decline of microbial diversity. During AD flares biofilm-growing *Staphylococcus aureus* emerges as the major colonizer in the skin lesions, in strict association with disease severity. The chronic production of inflammatory cytokines in the skin of AD individuals concurs at supporting *S. aureus* biofilm overgrowth at the expense of other microbial commensals, subverting the composition of the healthy skin microbiome. The close relationship between the host and microbial biofilm resident in the skin has profound implications on human health, making skin microbiota an attractive target for the therapeutic management of different skin disorders.

## 1. Introduction

Atopic dermatitis (AD) is the most common chronic inflammatory disease of the skin characterized by impaired epidermal barrier function, cutaneous inflammation, and dysbiosis of the cutaneous microbiota [1]. AD affects approximately 15–30% of children with 60% of cases occurring within a child’s first year and 85% before the age of 5 [2,3,4]. Several skin barrier defects have been described in AD, including an increased transepidermal water loss, a defective keratinocyte terminal differentiation, as well as a reduced level of ceramides, filaggrin, and antimicrobial peptides (AMP) [1,5,6]. In particular, mutations in the filaggrin gene (*FLG*) are considered as the strongest predisposing factor for AD [1,7,8]. However, most of the AD individuals do not have mutations in the *FLG* gene and 60% of mutation carriers do not show clinical signs of AD. Thus, *FLG* mutations alone are neither necessary nor sufficient to cause AD [7,9]. Growing evidence has highlighted the role of the cutaneous microbiota in the pathogenesis of AD and its complex interactions with the local host immune system [10]. Biofilm is the dominant mode of growth of the cutaneous microbiota supporting the stability of the resident microbial community and providing beneficial effects to the local and systemic host immunity [11,12,13]. Biofilm represents a survival strategy protecting the embedded cells from adverse environmental conditions, including antimicrobials and immune-mediated clearance (i.e., phagocytosis) [11,14,15]. Within the skin microbiota, coagulase-negative staphylococci (CoNS) represent the major colonizers known to compete with *Staphylococcus aureus* for the same ecological niche [13,16]. Alterations of the skin microbiota homeostasis, with the reduction in beneficial commensal microbes, significantly increase the risk of skin colonization by *S. aureus* [17]. The predominance of biofilm-growing *S. aureus* in AD lesions has been directly correlated to disease severity [18,19,20,21] and appears to be directly responsible for the occlusion of sweat ducts, skin inflammation, and pruritus [13,22,23,24,25]. This review highlights the basic mechanisms and the competitive dynamics involved in the homeostasis of the skin microbiota and the regulation of biofilm formation, focusing on the pathologic mechanisms promoting *S. aureus* colonization and chronic persistence in AD. 

## 2. Skin Microbiota Community in Healthy Skin and AD

Skin microbiota plays a key role in health and disease, by sustaining the epidermal barrier function, maintaining the immune homeostasis, and preventing the growth of pathogenic bacteria [26,27,28]. The analysis of skin microbiota was originally based on the culture method. Using this approach, the skin bacteria are collected by a swab and plated onto the appropriate growth media. Culture-based methods are primarily used for antimicrobial susceptibility testing, to analyze strain-specific virulence elements as well as genetic and proteomic bacterial profiles. Recent advances in DNA-sequencing techniques have allowed for the comprehensive study of the whole microbial population in native skin environments, providing a new approach to study the unculturable bacteria in complex communities [26,27,28,29]. Thus, both culture-based methods and microbiome analysis (Table 1) represent essential approaches to study and to fully characterize bacteria [30]. 

Molecular approaches have revealed that in healthy subjects, the most represented skin bacterial phyla are *Actinobacteria*, *Firmicutes*, *Proteobacteria,* and *Bacteroidetes*, arranged in different proportions according to the areas and the layers of the skin [31,32,33]. At the genus level, the cutaneous microbiota is mainly formed by *Staphylococcus*, *Propionibacterium*, *Corynebacterium*, and *Streptococcus* [34,35]. Sebaceous sites are dominated by staphylococci and propionibacteria, while corynebacteria prevalently colonize moist sites such as the antecubital fossa and interdigital spaces [26]. Independent from the skin site and the exposure to different environmental factors, the relative proportion of the different microbial species forming the cutaneous microbiota remains largely stable at the community level during the life span of each individual [21,33]. The skin microbiota of AD individuals presents marked abnormalities when compared with healthy subjects [10,36,37,38,39,40,41,42]. The lesional skin in AD is more frequently colonized by *S. aureus* than non-lesional skin, with a drastic reduction of microbial diversity [26,36,39,40]. Such alterations of microbial diversity in AD flares are only partially reverted by the medical treatment of eczema manifestations [10]. *S. aureus* colonization also increases in the skin of AD-susceptible individuals during the remission phase of flare episodes [43]. In addition to lesional skin, individuals with AD exhibit an altered microbiome composition in non-lesional skin and nose, suggesting that a more extensive modification of the microbial communities is present in atopic eczema [36,38,44]. 

*S. aureus* and *S. epidermidis* are the dominant species in untreated AD flares while they represent only a minority of bacterial communities in the skin of healthy individuals [36,37,38,39,40,41,42]. The significant increase of *S. epidermidis* during AD flares is thought to represent a putative compensatory mechanism aimed at limiting *S. aureus* colonization [28,45]. Indeed, *S. epidermidis* was found to be the most common producer of antimicrobials, including high levels of extracellular serine proteases, which prevent *S. aureus* epithelial surface adhesion [46,47]. In pediatric patients, metagenomic analysis of AD flares revealed greater *S. aureus* dominance in those individuals with more severe disease persisting at lower relative abundances as well as in the post-flare skin, while *S. epidermidis* abundance increased in patients with less severe AD [48]. The involvement of specific clonal lineages of *S. aureus* in AD individuals may represent a complicating and worsening factor for AD [38]. Although the number of studies describing the clonal distribution of *S. aureus* in AD is limited and the virulence determinants may be expressed differently in various strains, the clonal complex [CC] typing revealed the presence of a specific distribution of *S. aureus* in individuals with eczema [38,49,50,51,52]. Indeed, the CC1 represents the most common isolates followed by CC5, CC8, CC15, and CC45, while CC30 is more frequently isolated in healthy subjects [50,51,52]. In particular, CC1 is associated with severe forms of AD and it is the most abundant group detected on AD skin of individuals with the *FLG* gene mutations [38,49,52]. Phylogenetic analysis also demonstrated that in AD flares can be observed a clonal expansion of endogenous *S. aureus* strains, during a period of several weeks or months [48,52]. The intra-host genetic heterogeneity of *S. aureus* populations suggests the existence of a selective pressure supporting those strains being able to persist within the host and to resist treatments [48,52]. In contrast with the specific clonal distribution of *S. aureus*, *S. epidermidis* communities in AD individuals were composed of different strains belonging to diverse clades [29,48]. 

In addition, the inter-flare skin presents distinctive microbial signatures, characterized by the enrichment in *Streptococcus* and *Gemella* and a strong depletion in dermacocci and deinococci, as compared to normal healthy skin [43]. Dermacocci and deinococci, which belong to the order *Actinomycetales*, are common colonizers of the skin of healthy individuals, producing secondary metabolites with anti-inflammatory and anti-microbial properties [53]. 

Most changes in the skin microbiota composition correlate with *FLG* mutations, suggesting a possible association between microbial factors and host genetics [38,44]. Remarkably, at the level of non-lesional skin, an increased *S. aureus* colonization in AD patients with FLG mutations as compared with those which do not show any *FLG* mutations [38] was observed. Reduced *FLG* expression has also been correlated with an increased *S. aureus* colonization of epidermal skin models, and in children with AD, the presence of *FLG* mutations predisposed to recurrent skin infections [54,55]. Nevertheless, other reported that in infants with AD, *FLG* mutations and skin barrier dysfunctions were not associated with an increased *S. aureus* colonization in the vestibulum nasi and/or fauces [56]. In addition to *FLG* mutations, the skin barrier defects of AD individuals can also be attributable to a significant decrease of ceramides in the stratum corneum (SC) [57]. Ceramides are sphingolipid molecules acting as a water permeability barrier present in the intercellular spaces of the SC [58,59]. In adult AD skin, reduction in the amount of ceramide in the SC predisposes to inflammatory skin progression and increased *S. aureus* colonization [59,60,61,62]. The high susceptibility to cutaneous infections in AD skin is further promoted by the dysfunction of the outermost layer of the epidermis. Indeed, the application of emollient ointment to restore barrier function has been shown to improve the structure and functioning of the epidermal barrier, thus increasing the resistance to skin infections [63,64]. Skin barrier alterations allow for the entry of microbes into the dermis, promoting the colonization of previously inaccessible sites. The chronic inflammatory status, as well as the establishment of new ecological niches, may concur at disrupting the balance of the normal cutaneous microbiota and promoting the overgrowth of bacterial species, such as *S. aureus*, which are better fitted to adapt to the new environmental conditions. However, it is still unclear whether variations in the composition of skin microbiota, and in particular the overgrowth of *S. aureus*, precede the onset of AD by stimulating the immune system, or if the chronic inflammatory condition of the skin of AD individuals contributes to the disruption of the skin microbiota homeostasis and thus leading to AD. Different metabolites produced by bacteria within the gut microbiota might manipulate the local immune responses influencing immunity at distal sites in the so-called gut-skin axis [65,66,67,68]. Dysbiosis of the gut microbiome frequently precedes the onset of AD and correlates with an altered immune response [68]. Specifically, the percentage of *Clostridium difficile*, *Escherichia coli* colonizing the gut of AD individuals is higher than in healthy controls, while the proportion of *Bifidobacteria*, Bacteroidetes, and Bacteroides is reduced [65,69,70,71].

## 3. *Staphylococcus aureus* Biofilm Growth Cycle

Skin colonization by *S. aureus* primarily requires skin adhesion, which is followed by biofilm production [72]. The latter provides a tridimensional scaffold and a barrier against microbial competitors, antimicrobials, as well as the host innate immunity [11,13]. The attachment of *S. aureus* is mediated by host factors such as fibrinogen, fibronectin, and collagen by the surface proteins, referred to as microbial surface components recognizing adhesive matrix molecules (MSCRAMMs) [73]. The subsequent maturation of the biofilm occurs through cell division and requires the production of the extracellular polymeric matrix [13]. The production and composition of the biomass may vary between different *S. aureus* isolates, but in general, it is composed by host factors, the polysaccharide intercellular adhesin (PIA), proteins, extracellular DNA (eDNA) and amyloid fibrils [73,74,75,76]. PIA is a poly-β-(1-6)-N-acetylglucosamine, partially deacetylated, and charged positively [77,78]. The deacetylation of PIA is of great biological importance since it contributes to cellular adherence through electrostatic interactions [79]. The biosynthesis and accumulation of PIA on the bacterial surface are regulated by the intercellular adhesion ADBC (*ica*ADBC) locus. This mechanism was initially described in *S. epidermidis* and subsequently confirmed in *S. aureus* and many other staphylococcal species [80,81]. The biosynthesis of PIA comprises N-acetylglucosamine transferase (*ica*A and *ica*D), a deacetylase (*ica*B), an exporter (*ica*C), and a regulatory gene (*ica*R) [72,79]. Cells defective in synthesizing PIA produce less biomass than the wild-type strain, are less efficient in colonizing skin epithelial cells, more susceptible to antibacterial agents and the killing activity by polymorphonuclear leukocyte [79,82,83]. Most of *S. aureus* nosocomial and invasive isolates harbor the *ica*ADBC locus [81]. Prevalence studies of *ica*A, *ica*B, *ica*C and *ica*D genes in *S. aureus* showed a variable frequency, ranging from 50% to 75% for single genes, with about 50% of strains carrying the entire gene locus (*ica*ADBC) [84]. Notably, *S. aureus* strains isolated from the lesional skin of AD individuals were found to be positive for *ica* genes in more than 90% of cases [21,22,78]. Although PIA plays a central role in the process of *S. aureus* biofilm formation, numerous studies have shown that biofilm can also be formed in the absence of the *ica* operon by a PIA-independent mechanism [78]. PIA-independent mechanisms promote microbial attachment and biofilm formation through the activity of cell surface components such as teichoic acid, the cell wall-associated fibronectin-binding proteins A and B (FnBpA and FnBpB), which are particularly important for MRSA adhesion and biofilm formation, the autolysin extracellular DNA (eDNA), and the biofilm-associated protein (Bap) [85,86,87,88,89,90,91]. In particular, fibronectin and fibrinogen allow for the efficient binding of *S aureus* to the skin of individuals with AD but not to the skin of patients with other inflammatory dermatological diseases such as psoriasis or the normal skin of healthy subjects [19]. *S. aureus* secretes several proteins with adhesive properties, including the extracellular adherence protein (Eap), the extracellular matrix binding protein (Emp) or the protein beta toxin (Hlb), which interact with the eDNA in the biofilm matrix [92,93]. Nevertheless, the importance of individual proteins in the initial adhesion process and biofilm maturation varies largely between strains [94]. The PIA-independent mechanisms, however, appear important for host colonization and chronic persistence by allowing for a dynamic adaptation of the biofilm matrix in response to extracellular stimuli, being necessary to evade immune surveillance and to tolerate antibiotic treatments [78].

The detachment of *S. aureus* from the biofilm mass and its dispersal into the environment is mediated by the production of extracellular enzymes or surfactants controlled by the activity of the accessory gene regulator (*agr*) system [95,96,97]. The *agr* locus is a quorum sensing (QS) communication system regulated by small molecules called auto-inducing peptides (AIP) and influenced by cell density and environmental factors [98]. In *S. aureus*, AIP accumulates reaching a threshold level that determines the activation of the histidine kinase AgrC. The activated AgrC phosphorylates the regulator AgrA, which leads to the transcription of the *agr* operon and the subsequent production of RNA molecules (RNAIII) [99,100]. The *agr* controls different proteases, with the potential to degrade components of the biofilm matrix in vitro and in the skin and soft tissues of animal infection models [101,102,103]. Specifically, *agr* up-regulates several virulence determinants (toxins, proteases, lipases, nucleases), and down-regulate the expression of surface binding proteins. This responds to a specific time-dependent strategy of adaptation that requires the production of binding proteins during the initial stage of adhesion to host tissues, followed by the release of toxins and degradative exoenzymes when the colonization/infection is established, to acquire the necessary nutrients [100]. RNAIII, in turn, controls the expression of several genes, including the δ-toxin, and member the phenol-soluble modulins (PSMs) family [104,105]. PSMs appears to be of particular relevance in vivo, being capable of forming channels leading to the dispersal of bacterial cells from the biofilm matrix, thus promoting the dissemination of biofilm-associated microbial cells [106,107]. On the other hand, δ-toxin, which is produced at high levels by *S. aureus* strains, can induce mast cell degranulation, thus contributing to the severity of the inflammation primarily triggered by the IgE-mediated allergic response [108]. The use of the *agr*-inhibitor solonamide B (*solB*) in a mouse model of atopic skin, abolished δ-toxin production and significantly reduced the production of proinflammatory cytokines, reinforcing the notion of *S. aureus* direct participation to disease pathogenesis and, in turn, providing a possible therapeutic target potentially exploitable for the treatment of *S. aureus*-induced skin disorders [109]. 

## 4. *Staphylococcus aureus* Biofilm in the Pathogenesis of Atopic Dermatitis

The severity of AD is significantly associated with *S. aureus* colonization, which is prevalently represented in the form biofilm at the surface of AD skin [13,21,22,24]. *S. aureus* biofilm rapidly grows on epidermis, inducing hypoxia and damages in the protective epidermal barrier [110]. Skin barrier dysfunction is a distinctive hallmark of AD and defects in epidermal barrier promote the interaction of external antigens with skin-resident immune cells, thus exacerbating the local inflammation that, in some cases, might develop as a systemic response [111,112]. Filaggrin degradation products are responsible for pH regulation and hydration of the SC [111,112,113,114]. Null mutations in *FLG* reduce the levels of filaggrin degradation products in the skin, impairing the barrier function and affecting the composition and structure of the SC [7,115,116]. *S. aureus* can grow in a pH range between 5 and 9, but acidification mediated by filaggrin degradation products limits the growth rates and the adherence of *S. aureus* to keratinocytes [44,117,118,119]. The transcription of over 400 *S. aureus* genes appears to be differentially regulated by pH with marked differences in growth conditions at pH 5.5 versus those at pH 7.5 [119]. The acidic pH lowers the expression and activity of clumping factor B (ClfB) and fibronectin-binding protein A (FnBPA), which are involved in adherence and colonization of *S. aureus*, and that of molecules promoting immune evasion, such as the *S. aureus* protein A (SpA) [44,120]. Thus, alkalinization caused by the reduction in filaggrin and its breakdown products may favor *S. aureus* proliferation, adhesion, biofilm formation, and persistence of *S. aureus* in AD skin. 

*S. aureus* strains with a specific genetic background may contain combinations of virulence elements affecting the clinical outcome [121]. The most frequent CC detected in AD patients are CC1, CC5, CC15, and CC45 [38,50,51,52]. CC1 colonizes, in a significantly higher number, the skin of individuals with *FLG* mutations than *FLG* wild-type, whereas CC30 is more common in healthy individuals [36,51,52]. Notably, clonal lineages differ in their ability to form a biofilm. CC5, CC15, CC30, and CC45 were all able to produce biofilm, although at different levels [122]. In particular, the CC15 and CC45 strains produce a large amount of biofilm rapidly compared to CC5 and CC30 [122,123]. In terms of antibiotic resistance, it is important to note that methicillin-resistant strains preferentially colonized the skin of individuals with a less severe AD while methicillin-sensitive strains were primarily associated with the more severe AD [48]. This suggests that different strains of *S. aureus* with specific virulence elements may contribute to exacerbating skin inflammation as part of AD pathogenesis.

**Figure 1:**
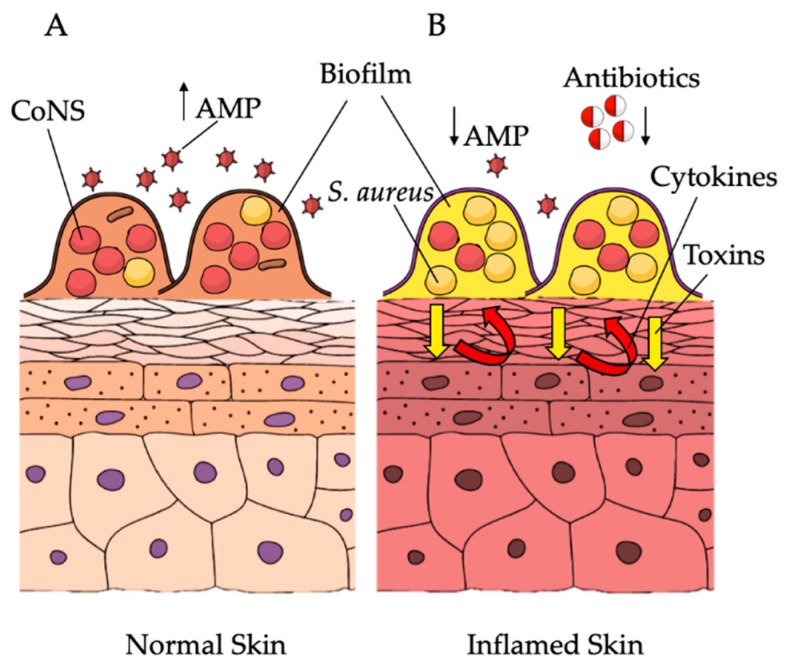
Skin microbiota variation and biofilm production in the pathogenesis of atopic dermatitis. (**A**) In the healthy skin coagulase-negative staphylococci (CoNS) compete with *Staphylococcus aureus* for the same ecological niche. (**B**) In AD, overexpression of inflammatory cytokines promotes *S. aureus* overgrowth, thus establishing *S. aureus* biofilm as the most abundant microorganism at the expense of other skin commensals. In turn, *S. aureus* participates in sustaining chronic inflammation in eczematous dermatitis. Arrows indicate increase (↑) and decrease (↓). The image is adapted from Mind the Graph (https://mindthegraph.com) under a Creative Commons License.

In vivo, the presence of *S. aureus* biofilm occludes sweat ducts in AD skin lesions, while this phenomenon is absent or much less recognizable in non-lesional areas [22,24,124]. As previously noted, most *S. aureus* strains, isolated from AD lesions, are strong biofilm producers in vitro and their ability to form biofilm was found to be significantly associated with the severity of the disease [21,22,124,125]. Biofilm-growing *S. aureus* can directly exacerbate AD severity (Figure 1), leading to refractory and recurrent infections, with increased resistance to the host immune responses and reduced susceptibility to antimicrobials when compared to their planktonic counterparts [13,21,22,24,126]. Clinical improvement in AD correlates with a reduction in *S. aureus* colonization [126,127,128]. However, although antibiotics and antiseptics can reduce *S aureus* colonization, recolonization occurs frequently within a few weeks, leading to limited clinical improvement and relapses of pathologic manifestations [129,130]. The serious limitation in treating biofilm-related infections is the higher concentration of antimicrobials required to kill bacterial cells, which can be hundreds of times higher than the minimum inhibitory concentration (MIC) assessed in planktonic culture [21,131,132,133]. Fusidic acid and mupirocin represent the most commonly used antibiotics for the treatment of cutaneous *S. aureus* colonization/infection, although the frequency of resistant isolates is increasing at an alarming rate [130]. Specifically, resistant strains to fusidic acid rose from 2% to 10%–38% [129,130]. Given the increased prevalence of antibiotic resistance and the lack of durable therapeutic benefits, short-term use of oral or topical antibiotics for the treatment of *S. aureus* colonization/infections has been suggested in place of the long-term use of systemic or topical antibiotics [134,135]. The chronic persistence and recurrent course of AD following antimicrobial therapy are strongly suggestive of the presence of a biofilm-associated colonization/infection. The efficacy of antimicrobials against planktonic and biofilm-growing *S. aureus* isolates from AD was found to depend mainly on the level of biofilm production. Weak biofilm producers gave equivalent susceptibility profiles when assessed in planktonic (minimum inhibitory concentration–MIC), or biofilm grow (biofilm MIC–BMIC) conditions [21]. Conversely, moderate/high biofilm producer strains showed a marked restriction of the effective antibiotic options as compared to those assessed by conventional MIC, and the difference correlated with the quantitative levels of biofilm production [21,136]. This could partially explain the conflicting data regarding the clinical benefit of anti-staphylococcal drugs in the treatment of a moderate and severe form of AD, even when the selection of the antibiotic was based on the MIC results [21,129,130,134,137,138,139,140]. In order to contain the risk of bacterial drug-resistance, in the absence of consistent evidence supporting a beneficial effect of antibiotic therapy, antiseptics have been proposed to reduce bacterial load. Sodium hypochlorite, which is of common use in AD, is effective against *S. aureus* biofilm [127,128]. Nevertheless, concentrations required for the removal of *S. aureus* biofilm were higher than those currently used in bleach baths for patients with AD [126].

*S. aureus* biofilms also showed reduced susceptibility to killing by AMPs, when compared with their planktonic counterpart [24]. Cathelicidin LL-37 is one of the most well-characterized AMPs and an important effector molecule of innate immunity in the skin [141,142]. It is present in the specific granules of neutrophils and keratinocytes and the local concentrations of this peptide increase in inflamed skin [143,144,145,146]. The negatively charged polysaccharides, comprising the extracellular matrix of *S. aureus* biofilm, interact with LL-37 [24]. *S. aureus* proteases present in the biofilm, such as staphopains, can degrade LL-37, generating smaller peptide fragments that modify or inactivate the AMP on both planktonic and biofilm bacteria [24,147]. Staphopain B degrades LL-37 into shorter peptide fragments, inactivating its bactericidal activity, though maintaining the potential to induce a pro-inflammatory response in AD skin [147]. Consequently, the degradation of LL-37 by staphopains may trigger and further amplify the inflammatory process in AD [24,147]. *S. aureus* biofilm formation is significantly inhibited by the presence of LL-37, whereas no or little inhibitory effect was found for the degradation products, which may disturb host defense, thus facilitating bacterial persistence [24]. Accordingly, high concentrations of LL-37 showed inhibitory effects on *S. aureus* biofilm production [148,149]. 

Though effective at decreasing skin colonization by *S. aureus*, antimicrobial treatment fails to restore the normal microbiome composition in AD. Conversely, topical treatments with corticosteroids, calcineurin inhibitors or moisturizing creams and emollients, which can contribute to reducing local inflammation and at restoring the skin barrier function, exhibit a positive effect on the composition of the skin microbiome [150,151,152]. Though closer to normal, the microbial composition of the treated individuals remains distinct from healthy controls [13]. In particular, local application of corticosteroids, even in the absence of antibiotic treatment, has been found to be effective at reducing *S. aureus* colonization through an undefined process [153,154,155]. Corticosteroids mainly exert an anti-inflammatory and immunosuppressive action, suggesting that the inflammatory milieu present in the AD lesions may play a role in promoting *S. aureus* colonization [21,156]. The immune-mediated inflammatory response in AD typically involves the prototypical cytokines IL-1β and IL-6 in the acute phase, switching to IFN-γ in the chronic phase [157]. IL-1β and IFN-γ were found to be capable of inducing a significant growth of *S. aureus* strains isolated from AD lesions, in a concentration-dependent manner [21]. Thus, inflammatory cytokines overexpressed in AD can stimulate the growth of biofilm-producing *S. aureus* during both the acute and chronic phases of the disease [21]. Such a growth-promoting activity appears to selectively support *S. aureus* at the expense of other bacterial skin commensals, suggesting that *S. aureus* might take advantage of an adaptive response to eukaryotic signals [21,36,40]. The molecular mechanisms associated with the growth response of *S. aureus* to different cytokines remain undefined, although others have reported a similar cytokine-induced growth response [156,158,159]. *Escherichia coli* displays chemoreceptors, with the potential to stimulate growth enhancement in the presence of different cytokines such as IL-2, IL-8, IL-15, and TNF-α [160,161,162,163]. Chemoreceptors on the surface of Gram-negative bacteria respond to proinflammatory cytokines modulating microbial virulence properties [163,164,165]. Specially, TNF-α and IFN-γ promote *E. coli* translocation in the gut while IL-8 induces transmigration across human lung epithelium in pulmonary infections. This suggests that different cytokines may represent chemoattractants for bacterial translocation [163,164,165]. *S. aureus* might exploit a similar process to that described for *E. coli* allowing skin colonization of AD individuals, which is chronically exposed to an inflammatory process. Further research is needed to clarify the mechanisms that support adhesion and chronic persistence of S. aureus on the skin of AD individuals, as well as the bacterial response to specific cytokines.

In AD flares, immune-induced cytokine production promotes *S. aureus* biofilm overgrowth. In turn, the overabundance of biofilm-growing *S. aureus* can directly stimulate the expression of proinflammatory cytokines, thymic stromal lymphopoietin, as well as the induction of the Th1/Th2 immune response [22,158,166]. It can directly penetrate the SC and epidermis, leading to the disruption of the skin immune homeostasis, thus potentiating the skin barrier defects already present on the skin of AD individuals [42]. *S. aureus* isolates recovered from the lesional skin of AD individuals produced high levels of delta-toxin which promotes mast cell degranulation and the release of Th2 type skin inflammatory molecules, including IgE production [167,168]. *S. aureus* biofilms have evolved strategies to ensure successful host colonization by evading the host’s innate immune system [169]. Macrophages are a major component of the inflammatory infiltrate in *S. aureus* colonization/infections. However, their penetration into the biofilm matrix is blocked by the robust fibrotic response [170]. Besides, *S. aureus* biofilm interferes with antimicrobial proinflammatory responses by promoting the enhanced recruitment of alternatively polarized macrophages with reduced anti-inflammatory properties, including reduced invasiveness [171,172,173]. The response guided by polarized macrophages exhibited poor microbicidal activity, a limited biofilm clearance potential, and increased fibrosis [174]. Moreover, secreted factors from *S. aureus* biofilm contribute to the significant reduction of the bactericidal activity and the proinflammatory responses of macrophages by activating the negative regulatory cascade of NF-kB pathway [175]. 

## 5. Competitive Interactions in the Skin Microbiota Biofilm in AD

**Figure 2:**
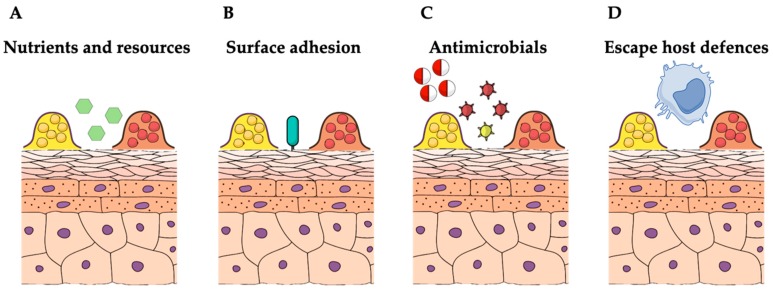
Competition dynamics among biofilm-growing staphylococci in the skin. (**A**) Staphylococci compete for the acquisition of the limited nutrients on the skin surface. (**B**) CoNS and *S. aureus* compete for surfaces attachment and persistent colonization. (**C**) Antibiotics and bacteriocins can limit staphylococci biofilm colonization. (**D**) *S. aureus* can contribute to the chronic inflammation in atopic dermatitis while escaping the immune system. The image is adapted from Mind the Graph (https://mindthegraph.com) under a Creative Commons License.

The skin is particularly poor in nutrients. Colonizing bacteria are subjected to strong environmental pressures, leading to the development of a large variety of strategies to limit other microbial competitors (Figure 2) [176,177]. The skin surfaces are constitutively colonized by CoNS, normally associated in biofilm communities [13,178]. Biofilm formation is considered a central factor in CoNS homeostatic control in the skin, allowing for colonization and persistence in almost all body surfaces, particularly on moist areas, such as nares, the axillae, inguinal, and perineal areas [13,178]. CoNS successfully adapt to their life as microbial biofilm communities, resident on the skin surfaces through the activation of numerous quorum sensing genes encoding for adhesion, biofilm production and AMP secretion [178]. *S. epidermidis* and *S. hominis* are the predominant CoNS species colonizing normal human skin with the potential to suppress inflammation, to stimulate the adaptive and innate immune system, and to produce molecules with antimicrobial activity against infectious pathogens [17,179,180,181,182,183,184,185]. CoNS and *S. aureus* share similar ecological niches, thus competing for surface adhesion and host colonization [17,46,92,185]. Polymicrobial colonization dynamics of CoNS species may alter *S. aureus* pathogenic behavior by influencing the host microbiota and stimulating the cutaneous immune system. Indeed, *S. epidermidis* can reduce inflammation after injury, regulate the development and influx of cutaneous T cells, and increase the expression of AMPs [181,182,183,186,187,188]. CoNS with bactericidal activity are abundant on the skin of normal population but rare on AD subjects [17]. Skin commensal-related factors play a key role in regulating the homeostasis of skin microbiota, and may directly affect the capacity of *S. aureus* to adhere and multiply on skin epithelia [17]. Thus, in addition to the growth promotion sustained by inflammatory cytokines, *S. aureus* colonization in AD may take advantage of the reduced proportion of “regulatory” skin commensals capable of exerting immune-modulatory and antimicrobial activities. *S. epidermidis*, *S. hominis*, *S. lugdunensis*, and *S. cohnii* are all capable of antimicrobial activities that inhibit the growth of *S. aureus* on the skin of both children and adults with AD [36,46]. The presence of *S. epidermidis* strains that secrete high levels of extracellular serine protease (Esp) can prevent nasal colonization by *S. aureus* [46,92]. Esp inhibits surface adhesions of *S. aureus* by degrading epithelial protein ligands and other proteins specifically required for biofilm formation [46,92]. Esp also enhances the susceptibility of *S. aureus* biofilms to immune system effector mechanisms [46,92]. For instance, the human beta-defensin 2 (hBD2), an AMP belonging to the human innate immune system and secreted by keratinocytes, exerts a low bactericidal activity against biofilm-growing *S. aureus* when acting alone. However, when hBD2 and Esp act in combination, they efficiently eradicate *S. aureus* biofilms [46]. *S. epidermidis* can also produce a mix of serine, cysteine, and metalloproteases, which specifically inhibits and destroys *S. aureus* biofilm [189]. This evidence indicates that protease production by *S. epidermidis* can provide a competitive advantage against *S. aureus* colonization and biofilm formation [46,189], although strain-specific differences were observed in *S. epidermidis* activity against *S. aureus* colonization on the lesional skin of AD individuals [17]. Further, some of the most effective bacteriocins produced by CoNS have the potential to limit *S. aureus* biofilms selectively [190]. Bacteriocins produced by *S. epidermidis* and *S. hominis* act synergistically with the human AMP LL-37 to potentiate their antimicrobial action towards skin pathogens, including *S. aureus* [17,179]. Human colonization of nares, axillae or groin with *S. lugdunensis* frequently correlates with a significant reduction of *S. aureus* colonization rate [191]. This competitive interaction may result from the production of lugdunin, a bacteriocin specifically active against *S. aureus*, by *S. lugdunensis* [185]. *S. warneri*, which is another member of the CoNS family, produces nukacin, a bacteriocin capable of bacteriostatic activity against planktonic *S. aureus*, although exerting a poor bactericidal activity against biofilms [192,193]. 

AD individuals also revealed a different composition in Gram-negative skin commensals when compared with healthy controls [194,195]. The culturable Gram-negative bacterial species *Roseomonas mucosa* isolated from healthy volunteers was shown to successfully reduce the growth of *S. aureus*, potentiating the skin barrier function and the innate immune response in AD [194,195]. These results suggest that specific strains of *R. mucosa* may influence AD, providing clinical benefit through multiple mechanisms that target epithelial barrier function, innate/adaptive immune balance, and *S. aureus* growth.

Different studies have also investigated the potential benefits of probiotics for the treatment of AD. Supplementation to pregnant women and infants at high risk of AD of *Lactobacillus rhamnosus* GG (LGG), *Bifidobacterium breve* M-16V, and *Bifidobacterium longum* BB536 reduced the incidence of AD in the probiotic-administered cases than the controls [196,197]. *Lactobacillus paracasei* NCC 2461 (ST11) was shown to increase the rate of barrier function recovery in a clinical study performed in women with sensitive skin [198]. Although promising, evidence supporting the use of probiotics for the prevention and treatment of AD is limited; its result is affected by multiple factors, such as the probiotic strain used, time and duration of administration, duration of exposure, and dosage [199].

## 6. Conclusions

**Figure 3:**
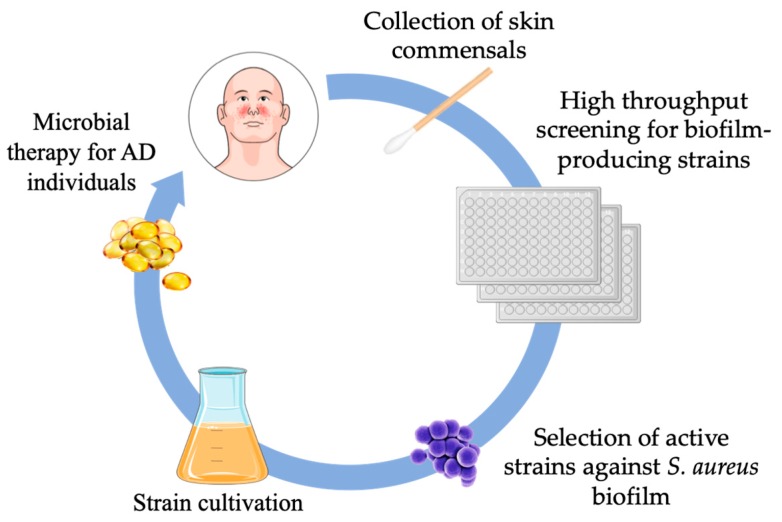
The exploitation of microbial dynamics in the skin may lead to new strategies for therapeutic interventions against pathogenic microbial biofilms. Initially, biofilm-producing skin commensal species isolated from AD individuals and capable of exerting an effective competition against *S. aureus* biofilm will be selected for expansion. The ideal strain(s) will be appropriately formulated for topical applications and administered to patients.

Skin microbiota represents a promising target for the treatment of dermatological diseases associated with microbial dysbiosis. In the skin, microbiota prevalently exists in the form of biofilm aggregates, in which competitive microbial interactions can influence the emergence and disappearance of microbial species. Skin commensals are also capable of actively competing with pathogenic bacteria by secreting antimicrobial factors that can impair adhesion and biofilm formation as well as modulate immune effector systems. AD is a multifactorial skin disease characterized by inflammatory lesions highly colonized by *S. aureus* biofilms, cutaneous inflammation, immune dysregulation and impaired epidermal barrier function [11,24,151]. *S. aureus* represents the major bacterial species in the skin lesions of individuals with AD and the degree of colonization significantly correlates with disease severity, suggesting that the same factors promoting such a dysbiosis may confer a selective advantage to *S. aureus*. The overrepresentation of *S. aureus* appears as the result of the growth-promoting activities exerted by inflammatory cytokines and a dramatic reduction in skin microbial diversity [17,21]. The high bacterial density and specific conditions that exist within *S. aureus* biofilm provide opportunities for cooperation as well as adaptation to a harsh and highly competitive environment [200,201]. Advancing knowledge of the biofilm communities and the dynamics regulating microbial competition within the cutaneous microbiota will provide useful tools for the development of novel therapeutic strategies for the treatment of skin disorders (Figure 3). To this end, transplantation of the skin microbiota from healthy individuals has been proposed as a promising option to restore the cutaneous microflora in AD [45]. Topical application of commensal microorganisms has been shown to reduce AD severity, suggesting a potential efficacy for this approach in limiting *S. aureus* colonization and restore the skin microbiota homeostasis [45,202]. At present, only a limited number of clinical trials with live bacteria, administered within a mix of topical probiotics, have shown a favorable outcome in AD [10,17,194,203,204]. However, such an approach, though feasible, should consider multiple factors, including the type of probiotic strains as well as time, duration and dosage of the treatment. Considering the great microbial diversity in the skin microbiota, it is also necessary to identify the specific risks associated with each probiotic strain and the risk factors connected with the host. CoNS are usually considered as harmless or even beneficial colonizers of the human skin, however, they cause opportunistic infections in the presence of host predisposing factors, such as indwelling medical devices or immunosuppression treatments [205]. In particular, *S. epidermidis* is emerging as a major nosocomial pathogen frequently associated with sepsis or biofilm-related infections on medical implants [205,206]. Antibiotic-resistant *S. epidermidis* are frequently isolated from patients and healthy individuals, suggesting that this bacterium may also play an important role as a source of resistance genes [205,207]. Besides, the contribution of direct bacterial interaction between skin colonizers and pathogens remains to be investigated.

Recent reports have demonstrated that commensal staphylococci, other than *S. aureus*, can potentially attenuate the development of AD in infants by modulating skin immunity [208,209]. Thus, the possibility to modify the skin microbiome at the level of biofilm communities with topical applications of selected skin commensals may represent a potential treatment for AD.

Further understanding of the mechanisms regulating *S. aureus* biofilms and the complex interactions of the microbial species resident in the skin will enable the development of prevention strategies and more targeted microbiota-based therapeutics in AD.

## Figures and Tables

**Table 1:** Summary of the previous studies of the skin microbiota in health and atopic eczema.

Techniques	Conclusion	Reference
Culture-Independent Method	Detection of bacteria within the dermis and dermal adipose of normal human skin	[30]
Culture-Independent Method	Despite the skin’s exposure to different environmental stressors, the microbial communities remain largely stable over time	[31]
Culture-Independent Method	*S. aureus* increases during AD flares and correlates with worsened disease severity	[34]
Culture-Independent Method	Differences in the skin microbiome between pediatric and adult with AD	[35]
Culture-Based Method	Increased *S. aureus* colonization in AD is associated with association with filaggrin gene mutations	[36]
Culture-Independent Method	Microbiome variation between affected and unaffected patients with AD before and after emollient treatment	[37]
Culture-Independent Method	*Staphylococcus*, *Pseudomonas*, and *Streptococcus* dominate the skin of AD individuals during flares	[38]
Culture-Independent Method	Dysbiosis and *S. aureus* colonization drives inflammation in AD	[39]
Culture-Independent Method	*S. aureus* can penetrate the stratum corneum and epidermis disrupting skin immune homeostasis	[40]
Culture-Independent Method	S. aureus increase in AD cohort over controls, in flares and non-flare skin of AD-susceptible individuals	[44]
Culture-Based Method	Alteration of sphingosine metabolism may predispose to increased *S. aureus* colonization in AD	[49]
